# Japanese-as-a-foreign-language acquisition affects native Chinese lexical processing among Chinese learners

**DOI:** 10.3389/fpsyg.2024.1457155

**Published:** 2025-01-07

**Authors:** Fengxiang Cai, Xiaodong Fei, Qichao Song

**Affiliations:** ^1^Faculty of Foreign Languages, Ningbo University, Ningbo, China; ^2^School of Japanese and International Studies, Beijing Centre for Japanese Studies, Beijing Foreign Studies University, Beijing, China

**Keywords:** Japanese-as-a-foreign-language acquisition, native Chinese lexical processing, orthographic similarity, phonological similarity, L2 lexical processing efficiency

## Abstract

Existing research has primarily focused on the influence of the native language on second language (L2) acquisition and processing, with less attention given to whether L2 acquisition affects native language processing. This study examines Chinese learners of Japanese, focusing on the orthographic and phonological similarities between two-character words in Chinese and Japanese. It investigates how these similarities affect native Chinese lexical processing at intermediate and advanced stages of Japanese learning and explores the predictive effect of L2 lexical processing efficiency on native language lexical processing efficiency at different stages of L2 learning. Through a comparison with native Chinese speakers who have not learned Japanese, the results indicate that L2 Japanese acquisition significantly impacts native Chinese lexical processing for Chinese learners of Japanese. Additionally, although there are some indications of the effects of orthographic and phonological similarities between Chinese and Japanese on native Chinese processing, the overall impact appears to be relatively weak. Moreover, the impact of L2 proficiency on lexical processing is influenced by L2 lexical processing efficiency, with both factors being closely related and jointly affecting lexical processing. Based on these results, this study proposes a potential pathway for native Chinese lexical processing among Chinese learners of Japanese.

## 1 Introduction

Different languages exhibit distinct orthographic and phonological characteristics, yet there is a certain degree of similarity between them. For instance, Dutch and English share similar orthographic and phonological systems, while Japanese and English demonstrate only phonological similarities. Due to these characteristics, language learners with different native languages (L1s) exhibit various features even when learning the same second language (L2). Previous studies have primarily focused on the influence of L1 on the acquisition and processing of an L2 (e.g., Dijkstra et al., 2000; Cai and Matsumi, 2009; Hsieh et al., 2017; Fei, 2019; Song et al., 2023), with little exploration of the backward transfer effects of L2 acquisition on L1 processing (e.g., Van Hell and Dijkstra, 2002; Boukrina and Marian, 2006; Chang, 2012; Bice and Kroll, 2015). By exploring the impact of L2 acquisition on L1 processing, we can investigate the characteristics of backward transfer (Kartushina et al., 2016) and analyze the mutual influence between the two languages from a reverse perspective. At the same time, understanding the complexities of language interaction in bilingual individuals informs cognitive models of language processing, illustrating how the learning of an L2 can reshape the processing and representation of the L1.

Japanese, which possesses both phonographic and ideographic writing systems, shares orthographic and phonological similarities with Chinese (e.g., Nakayama, 2002; Fei and Matsumi, 2012). It is generally believed that Chinese learners find it easier to learn Japanese compared to learners from non-Kanji backgrounds. However, studies indicate that Chinese learners also experience negative transfer effects from their L1 on L2 Japanese lexical processing (e.g., Cai and Matsumi, 2009; Fei, 2019; Fei et al., 2022). This raises an important question: does acquiring Japanese as a foreign language affect native Chinese processing? Currently, there is a lack of systematic research examining this question, particularly from the perspectives of orthography and phonology. Exploring the impact of L2 Japanese acquisition on native Chinese processing among Chinese-Japanese bilinguals sheds light on the influence of L2 acquisition on L1 from the perspective of ideographic writing systems.

Consequently, this study focuses on Chinese learners of Japanese to explore how L2 (i.e., Japanese) acquisition impacts L1 (i.e., Chinese) lexical processing. Specifically, we examine the effects of L2 acquisition on L1 processing through the lenses of orthographic and phonological similarities between the two languages and investigate how these effects are moderated by the learners’ Japanese proficiency and L2 lexical processing proficiency.

## 2 Literature review

### 2.1 Impact of L1 on L2 lexical processing

Lexical processing is regarded as one of the most important areas of study in cognitive psychology (Stella et al., 2024). The mental lexicon can be understood as a repository of lexical and conceptual representations, structured into organized networks that encompass semantic, phonological, orthographic, morphological, and other types of linguistic information (e.g., Wulff et al., 2019). In this mental lexicon, orthographic and phonological information is stored in the lexical representation, while semantic information is stored in the conceptual representation (Kroll and Steward, 1994; Aitchison, 2012). For L2 learners, how their L1 and L2s are stored in mental representations and whether they are separated or shared are issues of great interest. Since the 1950s, scholars have been exploring these questions. Through extensive empirical research, various models of mental representation, such as parallel, compound, and subordinate models, have been proposed (Weinreich, 1953). By the 1980s, the subordinate model gradually became the consensus, advocating for shared conceptual representations between bilinguals and independently stored lexical representations. Experimental results from Chen and Leung (1989) and Chen and Ng (1989) further validated this model, indicating that advanced English learners tend to use the concept mediation model for lexical processing, while beginner learners tend to use the lexical association model. During the process of L2 acquisition, as the learner’s L2 proficiency develops, the processing path of the mental lexicon also continues to change (e.g., Jiang, 2000; Zeng et al., 2022).

However, most of these studies have focused on comparisons between phonographic languages such as English (e.g., Kroll et al., 2010), with little focus on similar ideographic language systems like Chinese and Japanese. The biggest difference between Chinese and English lies in the relative independence between character shape and phonetic system in Chinese. In Chinese, a single character can correspond to multiple pronunciations. The character “行,” for example, can be pronounced as xíng (meaning “to walk” or “to function”) or háng (meaning “line” or “profession”), depending on the context. This reflects the more complex relationship between orthographic and phonological representations in the mental lexicon of Chinese. Nakayama (2002) emphasized the need to consider the attributes of vocabulary—namely orthographic and phonological similarities—to further explore the mental representation models in Chinese and Japanese. Numerous researchers have attempted to develop models of L2 lexical processing by analyzing the similarities between the two languages (e.g., Cai and Matsumi, 2009; Fei and Matsumi, 2012; Fei et al., 2022). These studies have demonstrated that the association between lexical representations influences not only the processing of orthographic information but also the processing of phonological information.

For instance, Matsushima and Fei (2011) investigated the effects of orthographic similarity between Chinese and Japanese on L2 Japanese processing. Their experimental materials consisted of Chinese characters categorized into five levels of orthographic differences (ranging from 0: identical, to 4: completely different; e.g., 0: 横 – 横, 4: 书 – 書), based on the similarity between Chinese characters and Japanese Kanji. Results from the word-naming task indicated no significant differences in response times for Kanji with orthographic differences of 0, 1, or 2, nor for those with differences of 3 and 4. However, response times for Kanji with differences of 0, 1, or 2 were significantly faster than those with differences of 3 or 4. These findings suggest that orthographic similarity between Chinese and Japanese bilinguals affects L2 Japanese processing.

Additionally, Cai and Matsumi (2009) utilized a cross-language priming task to examine lexical and conceptual representation models in Chinese and Japanese among advanced Chinese learners of Japanese. The results revealed the following pattern: for cognates with high orthographic similarity, such as “chair” (Chinese: 椅子 yǐ zi – Japanese: 椅子 isu), the orthographic representations are shared between the two languages. This leads to the simultaneous activation of both L2 and L1 during visual presentation, facilitating rapid access from orthographic representations to conceptual representations. In contrast, for non-cognates with low orthographic similarity, such as “program” (Chinese: 节目 jié mù – Japanese: 番組 bangumi), the orthographic representations are constructed independently for each language. This results in the sequential activation of L2 and L1 during visual presentation, which slows down access from orthographic representations to conceptual representations compared to cognates. These findings suggest that lexical representations in Chinese and Japanese may be shared or separated depending on the level of orthographic similarity, offering valuable insights into cross-linguistic interactions.

Moreover, Fei and Matsumi (2012), building on the concept of orthographic similarity, further explored the effects of phonological similarity between Chinese and Japanese on the auditory processing of L2 Japanese Kanji words among advanced Chinese learners. Through a lexical decision task, their results demonstrated that orthographic similarity positively facilitated semantic processing, while phonological similarity had an inhibitory effect. Specifically, words with high orthographic similarity but low phonological similarity [e.g., 学校 (school, Chinese: xué xiào, Japanese: gakkō)] showed the shortest response times, Whereas words with low orthographic similarity but high phonological similarity [e.g., 立派 (splendid, Japanese: lippa, which has no corresponding word in Chinese, but whose pronunciation is very similar to the Chinese characters ‘立 (lì)’ ‘派 (pài)’)] resulted in the longest response times.

The aforementioned findings have been validated through a series of examinations (Fei, 2013; Fei, 2015; Fei et al., 2022). Specifically, the influence of orthographic and phonological similarities on the auditory processing of Japanese Kanji words has been confirmed among intermediate to advanced learners, as well as learners of Japanese as a foreign language (JFL) and Japanese as a second language (JSL). These research findings demonstrate that during the processing of L2 Japanese vocabulary, orthographic similarity between Chinese and Japanese has a facilitating effect, while the influence of phonological similarity exhibits an inhibitory effect depending on the processing conditions (e.g., word-naming task, lexical decision task, or oral translation task).

In summary, regardless of whether the language is phonetic or ideographic, the L1 exerts a significant impact on L2 acquisition and processing. Among these influences, phonological similarity presents the most complexity, highlighting the difficulties Chinese learners encounter in acquiring the phonetics of Japanese vocabulary (Fei, 2013; Fei, 2015; Fei et al., 2022). This also raises an important research question: whether the phonetic acquisition of L2 Japanese affects the processing of L1, and if so, how it influences L1 processing.

### 2.2 Impact of L2 acquisition on L1 lexical processing

Compared to research on the impact of L1 on L2 acquisition and processing, there is relatively less focus on how L2 acquisition affects L1 processing. Van Hell and Dijkstra (2002) conducted a series of experiments with Dutch (L1)–English (L2)–French (L3) trilinguals to explore the influence of L2 knowledge on native language processing. The L1 stimulus words were either cognates with their translations in English (e.g., Dutch: ring, English: ring, French: bague), cognates with their translations in French (e.g., Dutch: stage, English: apprenticeship, French: stage), or non-cognates (e.g., Dutch: lente, English: spring, French: printemps). Participants completed a word association task or a lexical decision task in their L1. The results demonstrated that response times for cognates were significantly shorter than for non-cognates. However, this effect was observed only in individuals with higher proficiency in both L2 and L3. These findings suggest that L2 acquisition has a significant impact on L1 lexical processing, and this influence becomes stronger as L2 proficiency increases.

Additionally, Boukrina and Marian (2006) investigated the impact of phoneme overlap between L1 (Russian) and L2 (English) on bilingual lexical processing among Russian-English bilinguals. In their experiment, they did not use cognates, homophones, or homographs; instead, all words were unique to Russian and English. Their study, which employed an auditory lexical decision task, revealed an inhibitory effect of cross-linguistic phoneme overlap on L1 lexical processing. In other words, L1 words that shared phonology with L2 were processed slower and with less accuracy than words with unique native phonology. This finding aligns with Van Hell and Dijkstra’s (2002) demonstration of the significant impact of L2 acquisition on L1 lexical processing. Nonetheless, it is noteworthy that the effects observed in these two studies differ: Van Hell and Dijkstra found a facilitating effect, whereas Boukrina and Marian reported an inhibitory effect. This discrepancy may be attributed to factors such as the stimuli used, the sensory input modality (visual or auditory), and the linguistic distance between the languages studied.

Relevant evidence for this speculation can also be found in Kaushanskaya et al. (2011), which explored the impact of L2 acquisition on vocabulary and reading comprehension in L1 English among English-Spanish and English-Mandarin bilinguals. Results from vocabulary and reading tests, as well as self-assessments of proficiency, showed that higher L2 proficiency was associated with higher reading fluency in L1 among English-Spanish bilinguals. However, among English-Mandarin bilinguals, higher L2 proficiency was associated with lower reading fluency in L1. From these results, it can be inferred that the closer the linguistic distance between two languages, the higher the likelihood of a positive impact of L2 acquisition on L1 processing. Conversely, the greater the linguistic distance between the two languages, the more likely it is that L2 acquisition will have an inhibitory effect on L1 processing.

In summary, although some studies have focused on the effects of L2 acquisition on L1 processing, they have primarily concentrated on phonetic languages like English. Therefore, further research on ideographic languages, such as Chinese and Japanese, is needed. Building on the extensive research on the influence of L1 on L2 acquisition and processing, exploring the impact of L2 acquisition on L1 processing can provide deeper insights into the structure and function of mental representations in bilinguals. In particular, studying ideographic language systems such as Chinese and Japanese can offer new reference points for understanding language acquisition and processing.

### 2.3 Objectives and issues of this study

Having reviewed the relevant research findings, studies on the impact of L2 acquisition on L1 processing primarily encounter the following issues. First, the distance between languages plays a crucial role in investigating lexical processing models. Chinese and English have completely different orthographic and phonographic systems, while Chinese and Japanese share over 50% of Chinese characters (Japanese Kanji). Research on lexical processing across various language types enhances the study of lexical processing models for Chinese learners of different languages. However, most studies focus on distant language pairs, such as Chinese-English bilinguals, with few examining the psychological representation of the lexicon from the perspective of close-distance pairs, like Chinese-Japanese bilinguals. Second, while studies mainly explore the influence of L1 on L2 acquisition and processing, research on the impact of L2 acquisition on L1 processing is limited. Investigating the influence of L2 acquisition on L1 processing can clarify the correlation between bilingual languages and further analyze the backward transfer of L2 acquisition on L1. Third, existing research has established that the interaction between L2 and L1 is influenced by L2 proficiency (e.g., Van Hell and Dijkstra, 2002). This raises the question: Does proficiency similarly affect the interaction between Chinese and Japanese bilinguals? Additionally, among learners at the same proficiency level, does faster processing efficiency in their L2 further impact this interaction? These issues have not been systematically examined and warrant further investigation.

Based on the aforementioned considerations, this study aims to address the following two research questions:

RQ1: What is the impact of orthographic and phonological similarities between Chinese and Japanese on native Chinese lexical processing among Chinese learners of Japanese? Furthermore, does this impact vary according to proficiency in Japanese (L2 learning duration)?

RQ2: How is the impact of orthographic and phonological similarities between Chinese and Japanese on native Chinese lexical processing constrained by Japanese proficiency and L2 Japanese processing efficiency?

## 3 Materials and methods

### 3.1 Participants

In this experiment, a total of 58 participants took part (Mean age = 20.52, *SD* = 1.16; 33 female, 25 male), all from the same university in China. Among them, 18 were fourth-year students majoring in Japanese (hereafter referred to as J-N1), all of whom had achieved Japanese Language Proficiency Test N1 level (JLPT N1), indicating advanced proficiency. Another 20 participants were second-year students majoring in Japanese (hereafter referred to as J-N2), who had not taken the JLPT but were considered intermediate learners (approximately JLPT N2 level) based on their duration of Japanese study and program curriculum (Kouno and Hashimoto, 2016)^1^. Additionally, 20 Chinese native speakers (hereafter referred to as CC), majoring in non-foreign language disciplines at the university and using Mandarin Chinese exclusively in daily communication, also participated. After the experiment, all participants received compensation.

### 3.2 Experimental design

For Chinese learners of Japanese, mastering Japanese phonetics presents significant challenges (e.g., Fei et al., 2022; Geng et al., 2023). Therefore, acknowledging this aspect of L2 Japanese lexical processing, this study utilized a word-naming task that primarily emphasize phonological processing. Lexical decision tasks, on the other hand, pose difficulties in discerning whether participants are using their native Chinese or L2 Japanese to process lexical information, especially with homographs, which introduces ambiguity. In contrast, word-naming task requires output in the L1 and offer a clear distinction in processing based on L1 roots, thereby enabling precise investigation into how L2 Japanese influences L1 processing.

To address RQ1, which concerns the impact of vocabulary attributes and L2 proficiency on L1 lexical processing, the study examined the interaction between the levels of orthographic and phonological similarities between Chinese and Japanese and proficiency in the L2. As a comparative factor, L2 proficiency included the condition of being a Chinese native speaker with no knowledge of Japanese.

To address RQ2, which delves further into the influence of vocabulary attributes on L1 lexical processing constrained by L2 Japanese proficiency and processing efficiency, the study examined the three-way interaction among levels of orthographic and phonological similarities between Chinese and Japanese, L2 proficiency, and L2 lexical processing efficiency.

### 3.3 Experimental materials

Orthographic similarity is generally a physical variable, not subject to subjective change by learners, whereas phonological similarity is a psychological variable, with evaluations that may vary among different learners (e.g., Matsushima and Fei, 2011). Therefore, when discussing the influence of orthographic and phonological similarities, experimental materials cannot be selected based on a single standard. Based on the linguistic characteristics of Chinese and Japanese and the activation mechanism of psychological representations, phonological similarity between Chinese and Japanese was determined by comparing the pronunciation of a specific Japanese word to the pronunciation of the corresponding Chinese word (Tome et al., 2012). For Chinese and Japanese heterographic words, the Japanese pronunciation does not correspond to an existing Chinese word. Consequently, when the experimental material included a heterographic word, the Japanese word was translated into its Chinese equivalent for use in the experiment [e.g., 泥棒 (dorobō, thief) has no equivalent in Chinese; therefore, 小偷 (xiǎo tōu, thief) is used].

Based on the special relevance between Chinese and Japanese, this experiment strictly controlled the experimental materials and explored the influence of L2 acquisition on L1 processing in different groups. Since we employed a word-naming task with a primary focus on the effects of orthographic and phonological similarities, we controlled for potential semantic influences by ensuring that all selected words were Chinese-Japanese synonyms. Given the aforementioned considerations, the following determinations of orthographic and phonological similarities between Chinese and Japanese were made:

Orthographic similarity: Matsushima and Fei (2011) found that characters with a difference score of 2 or less (i.e., 0, 1, 2) were processed similarly by Chinese learners, indicating no significant difference. In other words, Chinese characters with a difference score of 2 or less are considered to have high orthographic similarity. Based on these findings and considering the distinction between compound and single-character words, we also followed Fei (2019)’s material selection criteria to identify words with high orthographic similarity, while heterographs were classified as having low orthographic similarity.

Phonological similarity: judgments were based on materials from Tome et al. (2012), who recorded the standard Mandarin pronunciation of Chinese vocabulary words and the standard Japanese pronunciation of the corresponding Japanese words. Chinese university students with no background in Japanese rated the similarity using a 7-point Likert scale (1: Not similar at all; 7: Extremely similar).

We selected a total of 45 words (with each group consisting of 15 Chinese words). The experimental materials comprised three conditions of Chinese words: Condition 1: high orthographic similarity; high phonological similarity. Condition 2: high orthographic similarity; low phonological similarity. Condition 3: low orthographic similarity; low phonological similarity. Additionally, 45 corresponding Japanese words were selected to address RQ2.

There were no significant differences in the frequency of usage across the three conditions [Chinese (referencing *BLCU Corpus,* Xun et al., 2016): *F* (2, 42) = 0.56, *p* = 0.576; Japanese (referencing *the Balanced Corpus of Contemporary Written Japanese (BCCWJ),* Maekawa et al., 2014): *F* (2, 42) = 0.16, *p* = 0.849]. The materials ensured that the phonological similarity was significantly higher in the high phonological similarity condition compared to the low phonological similarity condition, with no significant differences among materials in the low phonological similarity condition [*F* (2, 42) = 111.14, *p* < 0.001, multiple comparison results obtained using Tukey’s HSD correction: Condition 1 > Condition 2 [*t* (42) = 12.39, *p* < 0.001], Condition 1 > Condition 3 [*t* (42) = 13.37, *p* < 0.001], Condition 2 = Condition 3 [*t* (42) = 0.98, *p* = 0.593]]. To further ensure the homogeneity of the three conditions of Japanese stimuli, 22 s-year and 21 fourth-year Japanese students (who did not participate in the formal experiment and were matched in proficiency with participants) were asked to evaluate the familiarity of 45 Chinese and Japanese words using a 7-point Likert scale (1: Not familiar at all; 7: Extremely familiar). The results indicated no significant differences among the conditions for both second-year [*F* (2, 42) = 0.63, *p* = 0.540] and fourth-year students [*F* (2, 42) = 1.83, *p* = 0.173]. Details and examples of the experimental materials are presented in Table 1.

**Table 1:** Experimental materials’ indices and examples.

Condition	Phonological similarity	Log-transformed frequency		Japanese familiarity		Examples	
		Chinese	Japanese	N1	N2	Chinese	Japanese
1	5.33 (1.00)	4.60 (0.43)	3.81 (0.37)	6.77 (0.16)	6.63 (0.28)	椅子 (yǐ zi)	椅子 (isu)
2	2.02 (0.56)	4.81 (0.63)	3.78 (0.57)	6.65 (0.21)	6.64 (0.29)	学校 (xué xiào)	学校 (gakkō)
3	1.76 (0.55)	4.68 (0.56)	3.71 (0.51)	6.61 (0.31)	6.53 (0.28)	小偷 (xiǎo tōu)	泥棒 (dorobō)

Results are shown as mean (standard deviation). Condition 1: high orthographic similarity, high phonological similarity. Condition 2: high orthographic similarity, low phonological similarity. Condition 3: low orthographic similarity, low phonological similarity.

### 3.4 Experimental procedure

**Figure 1:**
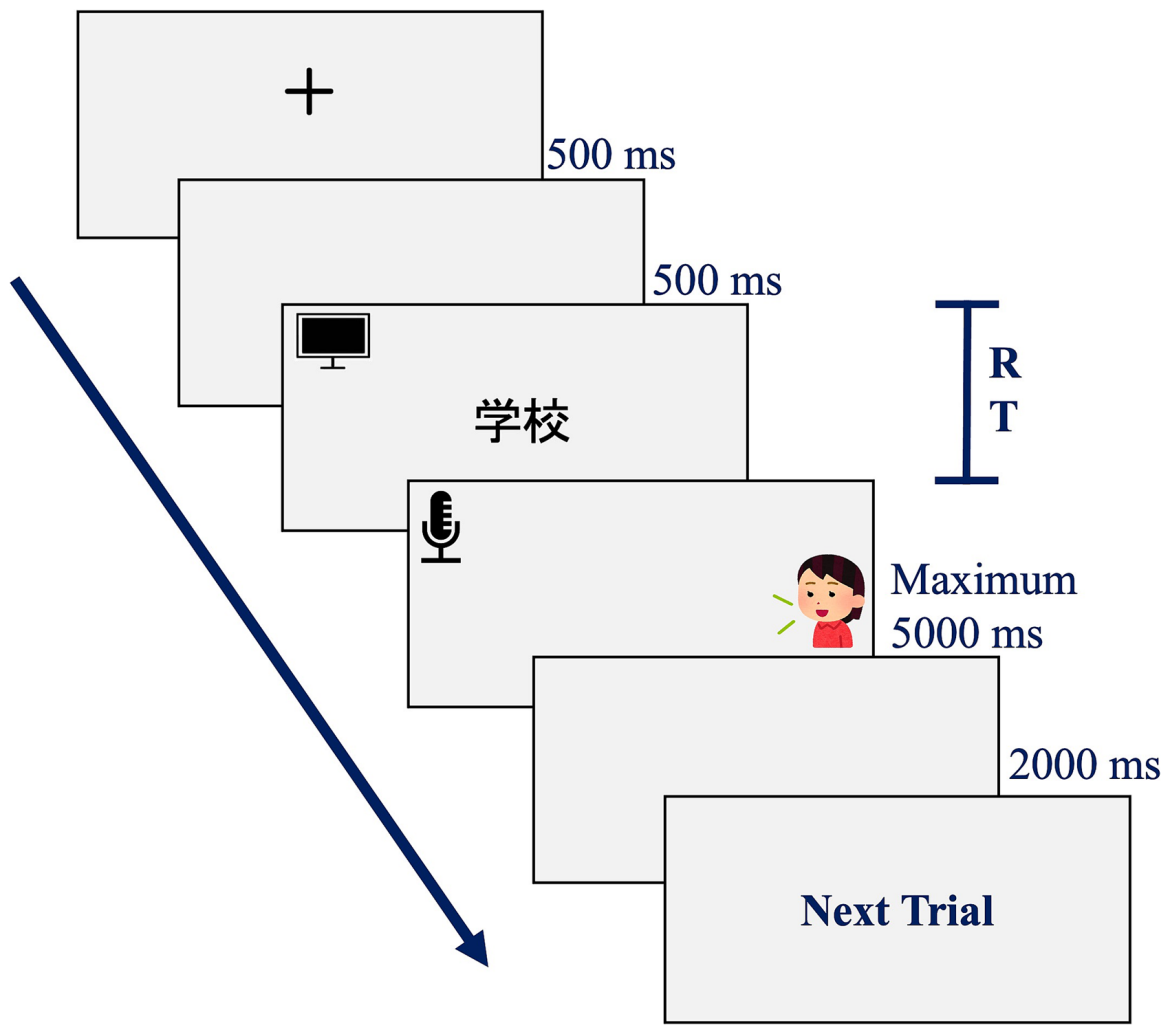
Experimental procedure.

The experimental program was developed using SuperLab Pro (ver. 4.0). The experimental procedure is depicted in Figure 1. Initially, a “+” symbol appeared on the computer screen for 500 ms, signaling that participants would soon see a word. Participants were instructed to quickly and accurately pronounce the Chinese words presented on the screen as soon as they appeared. Response times were automatically recorded by a voice key. If a participant failed to pronounce a word within the specified timeframe (5,000 ms), the word would disappear automatically.

One week later, the same group of participants, now learners of Japanese, engaged in a Japanese word-naming task. They were required to quickly and accurately pronounce the Japanese words displayed on the computer screen upon seeing them. Similarly, response times were recorded by the voice key. If a participant failed to pronounce a word within the specified timeframe (5,000 ms), the word would automatically disappear. All experimental stimuli were presented randomly.

## 4 Results and discussion

### 4.1 Data manipulation and analysis method

We excluded unresponsive data from the Chinese word-naming task (CC: 22 instances; J-N2: 17 instances; J-N1: 26 instances; removal rate: 2.49%) and the Japanese word-naming task (J-N2: 103 instances; J-N1: 45 instances; removal rate: 8.66%). Extreme response times, defined as very short (< 200 ms) or very long (> 3,500 ms), were also excluded (4 instances; removal rate: 0.09%). Additionally, inaccurate response data were excluded: no instances were removed from the Chinese word-naming task across the three groups, while 45 instances were removed from J-N2 and 6 instances from J-N1 in the Japanese word-naming task (removal rate: 2.98%). After the data cleaning process, response times were log-transformed to address skewness.

As previously mentioned, this study employed a visually presented word-naming task to explore the effects of orthographic and phonological similarities between Chinese and Japanese bilinguals. To control for potential semantic influences, we ensured that the materials in all three conditions shared the same meanings in both Chinese and Japanese, thereby excluding homographs (i.e., items with high orthographic similarity but low semantic similarity). For example, homographs such as ‘用意,’ which means ‘intent’ in Chinese (yòng yì) but ‘preparation’ in Japanese (yōi), were not included. This constraint made it difficult to include both orthographic and phonological similarities in a single model for statistical analysis.

Consequently, to prevent the two factors from interacting and compromising the objectivity of the experimental results, the study examined the impact of orthographic and phonological similarities between Chinese and Japanese separately on native Chinese lexical processing. Specifically, to investigate the impact of orthographic similarity and Japanese proficiency levels, the response-time data from Condition 2 (*high* orthographic similarity; low phonological similarity) and Condition 3 (*low* orthographic similarity; low phonological similarity) were analyzed statistically. To investigate the impact of phonological similarity and Japanese proficiency levels, the response-time data from Condition 1 (high orthographic similarity; *high* phonological similarity) and Condition 2 (high orthographic similarity; *low* phonological similarity) were analyzed statistically. The mean response times and standard deviations for each condition are presented in Tables 2, 3.

**Table 2:** Mean response times (ms) and standard deviations under conditions of orthographic similarity and Japanese proficiency levels.

	Orthographic similarity	CC	J-N1	J-N2
Chinese word-naming task	Low	612.09 (93.07)	656.55 (112.36)	676.58 (126.74)
	High	610.12 (96.45)	662.00 (115.83)	701.55 (167.42)
Japanese word-naming task	Low	–	1012.83 (378.23)	1218.60 (421.46)
	High	–	1023.32 (375.95)	1213.38 (459.76)

Results are shown as mean (standard deviation).

**Table 3:** Mean response times (ms) and standard deviations under conditions of phonological similarity and Japanese proficiency levels.

	Phonological similarity	CC	J-N1	J-N2
Chinese word-naming task	Low	610.12 (96.45)	662.00 (115.83)	701.55 (167.42)
	High	609.00 (91.38)	692.09 (210.54)	686.61 (153.79)
Japanese word-naming task	Low	–	1023.32 (375.95)	1213.38 (459.76)
	High	–	932.85 (344.98)	1083.60 (357.18)

Results are shown as mean (standard deviation).

Data analysis for RQ1 (Section 4.2) was performed using R software (ver. 4.2.1, R Core Team, 2022), employing linear mixed-effects modeling with the *lme4* package (Bates et al., 2015) and the *lmerTest* package (Kuznetsova et al., 2017). The *emmeans* package (Lenth, 2022) was utilized to investigate interactions. The model with the lowest Akaike Information Criterion (AIC) was chosen as the optimal model for fitting. Random effects in the model included both random slopes and intercepts. If the model with the lowest AIC did not converge, we further simplified the random effects model. The *anova* function is used to perform an omnibus test on the optimal model. Data analysis for RQ2 (Section 4.3) was performed using Jamovi software (ver. 2.3, The Jamovi Project, 2022), and included correlation analysis and general linear model analysis.

### 4.2 Analysis results and discussion for RQ1

In this section, we discuss the influence of orthographic and phonological similarities between Chinese and Japanese on native Chinese processing. By comparing the performance of Chinese native speakers with no knowledge of Japanese to that of Chinese learners of Japanese (J-N1 and J-N2), we first determine whether native Chinese processing is affected by the L2 (Japanese). We then explore whether this influence is moderated by Japanese proficiency, as indicated by the duration of Japanese learning.

#### 4.2.1 Impact of orthographic similarity and Japanese proficiency

The model selection results show that the optimal model was [logRT ~ orthographic × J-pro + (1 | participant) + (1 | item)]. The results of the linear mixed-effects model analysis indicate that the main effect of orthographic similarity was not significant [*F* (1, 29.48) = 0.45, *p* = 0.508]. However, the main effect of Japanese proficiency levels was significant [*F* (2, 58.00) = 5.36, *p* = 0.007]. Multiple comparisons using Tukey’s HSD correction reveal that response times for CC were significantly faster than those for J-N1 learners (*β* = 0.03, se = 0.02, *df* = 60.80, *t* = 2.01, *p* = 0.049) and J-N2 learners (*β* = 0.05, se = 0.02, *df* = 60.80, *t* = 3.15, *p* = 0.003). Although J-N1 learners were slightly faster than J-N2 learners, the difference was not statistically significant (*β* = 0.02, se = 0.02, *df* = 60.80, *t* = 1.06, *p* = 0.295). The interaction effect between orthographic similarity and Japanese proficiency levels was not significant [*F* (2, 1619.26) = 2.16, *p* = 0.112].

#### 4.2.2 Impact of phonological similarity and Japanese proficiency

The model selection results show that the optimal model was [logRT ~ phonological × J-pro + (1 | participant) + (1 | item)]. The results of the linear mixed-effects model analysis indicate that the main effect of phonological similarity was not significant [*F* (1, 29.50) = 0.06, *p* = 0.805]. However, the main effect of Japanese proficiency levels was significant [*F* (2, 57.97) = 6.30, *p* = 0.003]. Multiple comparisons using Tukey’s HSD correction show that CC had significantly faster response times compared to J-N1 learners (*β* = 0.04, se = 0.02, *df* = 60.50, *t* = 2.56, *p* = 0.013) and J-N2 learners (*β* = 0.05, se = 0.02, *df* = 60.60, *t* = 3.32, *p* = 0.002). Although J-N1 learners had faster response times than J-N2 learners, there was no significant difference between them (*β* = 0.01, se = 0.02, *df* = 60.50, *t* = 0.67, *p* = 0.504).

The interaction effect between phonological similarity and Japanese proficiency levels was significant [*F* (2, 1068.42) = 4.49, *p* = 0.011]. Simple main effects analysis results indicate that: (1) Phonological similarity did not have a significant effect at any proficiency level (CC: *β* = 0.00, se = 0.01, *df* = 46.40, *t* = 0.01, *p* = 0.995; J-N1 learners: *β* = 0.01, se = 0.01, *df* = 49.10, *t* = 1.43, *p* = 0.158; J-N2 learners: *β =* 0.01, se = 0.01, *df* = 46.40, *t* = 0.80, *p* = 0.429). (2) Under conditions of high phonological similarity: CC had significantly faster response times compared to J-N1 learners (*β* = 0.05, se = 0.02, *df* = 67.60, *t* = 2.92, *p* = 0.005) and J-N2 learners (*β* = 0.05, se = 0.02, *df* = 67.70, *t* = 2.99, *p* = 0.004). There was no significant difference between J-N1 learners and J-N2 learners (*β* = 0.00, se = 0.02, *df* = 67.60, *t* = 0.01, *p* = 0.991). (3) Under conditions of low phonological similarity: CC had significantly faster response times compared to J-N1 learners (*β* = 0.03, se = 0.02, *df* = 67.30, *t* = 2.07, *p* = 0.042) and J-N2 learners (*β* = 0.05, se = 0.02, *df* = 67.30, *t* = 3.48, *p* < 0.001). There was no significant difference between J-N1 learners and J-N2 learners, but the response-time difference increased (*β* = 0.02, se = 0.02, *df* = 67.20, *t* = 1.32, *p* = 0.191).

#### 4.2.3 Discussion for RQ1

The results indicate that, regardless of whether the orthographic and phonological similarities were high or low, Chinese speakers who had not studied Japanese exhibited the highest processing efficiency. This confirms that the acquisition of L2 Japanese vocabulary can interfere with native Chinese processing, further enriching existing empirical research (Linck et al., 2009; Alario et al., 2010) from the perspective of Chinese-Japanese bilinguals and demonstrating that L2 acquisition can affect L1 processing. Although the above analyses showed that Japanese proficiency (i.e., learning duration) did not significantly affect native Chinese processing, the descriptive statistics revealed a trend whereby intermediate learners had longer response times compared to advanced learners. This suggests that Japanese proficiency may have some influence, albeit a relatively minor one. This could be attributed to the characteristics of the participants in this study, who primarily used Chinese outside the classroom despite learning Japanese in class, resulting in lower Japanese usage frequency.

Additionally, while orthographic similarity did not show a significant interaction effect with Japanese proficiency, a significant interaction effect between phonological similarity and Japanese proficiency was revealed. Specifically, when phonological similarity was high, the response-time difference between J-N2 and J-N1 learners was small. In contrast, under low phonological similarity, L1 processing efficiency for J-N1 learners showed a certain degree of improvement. This result supports the notion that phonological similarity can negatively impact lexical processing (e.g., Fei, 2019; Fei et al., 2022), and this study further demonstrates that the impact of phonological similarity may be more pronounced for J-N1 learners. This indicates that vocabulary with high phonological similarity between Chinese and Japanese may negatively affect phonological processing in the L1 due to the similar phonological information.

In summary, the analysis results confirm that the acquisition of L2 Japanese vocabulary significantly impacted native Chinese processing, with phonological similarity presenting a moderate inhibitory effect, while the effects of orthographic similarity were limited.

### 4.3 Analysis results and discussion for RQ2

In this section, we further explore whether the impact of L2 acquisition on native Chinese lexical processing is influenced by L2 processing efficiency. Specifically, building on the analysis from Section 4.2, we conduct a correlation analysis of the response times of Chinese words corresponding to Japanese words based on Japanese proficiency levels. Furthermore, we incorporate the standardized response times of Japanese word-naming task into a general linear model to examine the predictive power of L2 processing efficiency.

#### 4.3.1 Impact of orthographic similarity, Japanese proficiency, and L2 processing efficiency

First, correlation analyses were conducted separately to assess the relationship between response times in L2 Japanese lexical processing and Chinese lexical processing across different levels of Japanese proficiency. At the J-N1 level, there was no significant correlation observed between Chinese and Japanese lexical processing efficiency (*r* = 0.09, *df* = 499, *p* = 0.052). Conversely, at the J-N2 level, a significant but low-level correlation was found between Chinese and Japanese lexical processing efficiency (*r* = 0.22, *df* = 513, *p* < 0.001).

**Figure 2:**
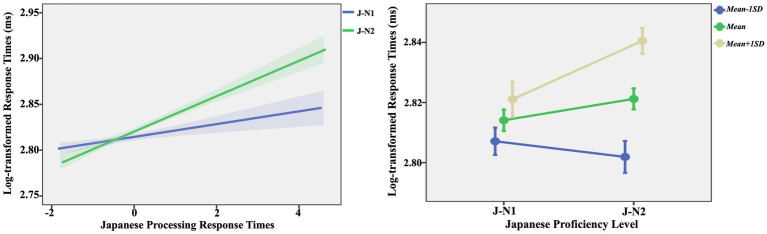
Interaction between Japanese proficiency and L2 processing efficiency in section 4.3.1.

Second, a general linear model was employed to investigate the predictive power of Japanese processing efficiency on native Chinese processing. The model included orthographic similarity, Japanese proficiency level, standardized Japanese lexical processing response times, and their interactions as independent variables, with log-transformed Chinese lexical processing response times as the dependent variable. The analysis results reveal the following:

1. The predictive effects of L2 processing efficiency were significant [*F* (1, 1,006) = 26.97, *p* < 0.001], indicating that longer Japanese lexical processing response times were associated with longer Chinese lexical processing response times.
2. The interaction between Japanese proficiency and L2 processing efficiency was significant [*F* (1, 1,006) = 5.93, *p* = 0.015]. Simple slope analysis results (Figure 2) indicate that for J-N1 learners, there was a significant trend of L2 processing efficiency predicting Chinese lexical processing efficiency (*β* = 0.01, se = 0.00, *df* = 1,006, *t* = 1.82, *p* = 0.069), suggesting that higher L2 Japanese lexical processing efficiency was associated with an increased tendency for Chinese lexical processing efficiency. For J-N2 learners, L2 processing efficiency significantly predicted Chinese lexical processing efficiency (*β* = 0.02, se = 0.00, *df* = 1,006, *t* = 5.85, *p* < 0.001), indicating that higher L2 Japanese lexical processing efficiency was associated with higher Chinese lexical processing efficiency. However, under conditions of high L2 processing efficiency (i.e., shorter response time, *Mean* − 1 *SD*), there was no significant difference in Chinese lexical processing efficiency between J-N2 learners and J-N1 learners (*β* = 0.01, se = 0.01, *df* = 1,006, *t* = 0.75, *p* = 0.456). Conversely, under conditions of low L2 processing efficiency (i.e., longer response time, Mean + 1 *SD*), Chinese lexical processing efficiency for J-N2 learners was significantly lower than that for J-N1 learners (*β* = 0.02, se = 0.01, *df* = 1,006, *t* = 2.69, *p* = 0.007).
3. The main effects of orthographic similarity [*F* (1, 1,006) = 1.03, *p* = 0.309] and Japanese proficiency [*F* (1, 1,006) = 2.03, *p* = 0.154], the interaction between orthographic similarity and Japanese proficiency [*F* (1, 1,006) = 0.64, *p* = 0.424], the interaction between orthographic similarity and L2 processing efficiency [*F* (1, 1,006) = 0.04, *p* = 0.842], and the three-way interaction among orthographic similarity, Japanese proficiency, and L2 processing efficiency [*F* (1, 1,006) = 0.57, *p* = 0.451] were all non-significant.

#### 4.3.2 Impact of phonological similarity, Japanese proficiency, and L2 processing efficiency

First, correlation analyses were conducted separately for L2 Japanese lexical processing response times and Chinese lexical processing response times based on different levels of Japanese proficiency. The results revealed that at the J-N1 level, there was no significant correlation between Japanese and Chinese lexical processing response times (*r* = 0.04, *df* = 491, *p* = 0.409). However, at the J-N2 level, a significant but low-level correlation was found between Japanese and Chinese lexical processing response times (*r* = 0.25, *df* = 507, *p* < 0.001).

**Figure 3:**
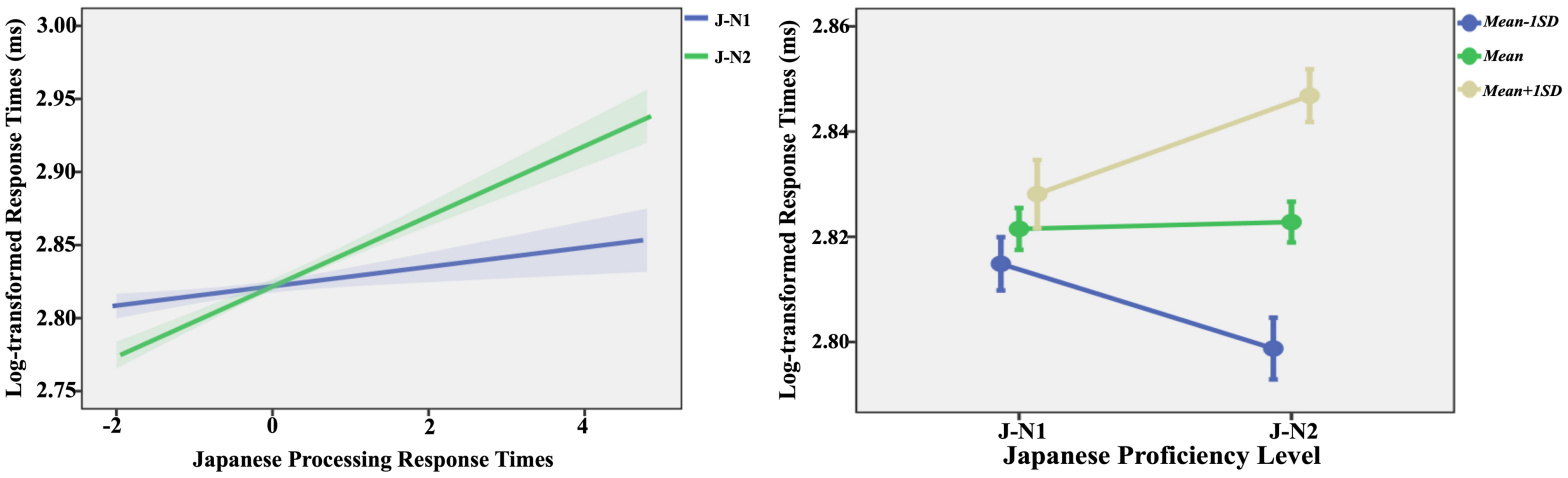
Interaction between Japanese proficiency and L2 processing efficiency in section 4.3.2.

Second, Japanese proficiency, phonological similarity, standardized Japanese lexical processing response time, and their interactions were included as independent variables in the model, with the log-transformed Chinese lexical processing response time as the dependent variable. The analysis results reveal the following:

1. The predictive effect of L2 processing efficiency was significant [*F* (1, 994) = 28.70, *p* < 0.001], indicating that a longer L2 Japanese lexical processing response time was associated with a longer Chinese lexical processing response time.
2. The interaction between Japanese proficiency and L2 processing efficiency was significant [*F* (1, 1,006) = 9.25, *p* = 0.002]. Simple slope analysis results (Figure 3) reveal that the L2 processing efficiency of J-N1 learners did not significantly predict their Chinese lexical processing efficiency (*β* = 0.01, se = 0.00, *df* = 994, *t* = 1.56, *p* = 0.118); as the L2 processing efficiency of J-N2 learners increased, their Chinese lexical processing efficiency also increased (*β* = 0.02, se = 0.00, *df* = 994, *t* = 6.24, *p* < 0.001). However, under conditions of high L2 processing efficiency, the Chinese processing efficiency of J-N2 learners was faster than that of J-N1 learners (*β* = 0.02, se = 0.01, *df* = 994, *t* = 2.08, *p* = 0.038); conversely, under conditions of low L2 processing efficiency, the Chinese processing efficiency of J-N2 learners was slower than that of J-N1 learners (*β* = 0.02, se = 0.01, *df* = 994, *t* = 2.28, *p* = 0.023).
3. The main effects of orthographic similarity [*F* (1, 994) = 1.62, *p* = 0.202] and Japanese proficiency [*F* (1, 994) = 0.05, *p* = 0.815], the interaction between orthographic similarity and Japanese proficiency [*F* (1, 994) = 1.96, *p* = 0.161], the interaction between orthographic similarity and L2 processing efficiency [*F* (1, 994) = 1.36, *p* = 0.244], and the three-way interaction among orthographic similarity, Japanese proficiency, and L2 processing efficiency [*F* (1, 994) = 0.13, *p* = 0.715] were all non-significant.

#### 4.3.3 Discussion for RQ2

Building on the analysis of RQ1, this section further explores whether the L2 Japanese processing efficiency of learners influenced their native Chinese processing efficiency. The results indicate that L2 processing efficiency significantly predicted L1 processing efficiency, with the impact being more pronounced for J-N2 learners. The descriptive statistics (Tables 2, 3) and *t-test* results (see text footnote 1) indicate that as learners’ Japanese proficiency improved, their L2 Japanese processing efficiency increased overall, while the predictive power of L2 processing efficiency on L1 lexical processing weakened.

The impact of orthographic and phonological similarities and their interaction with other factors were not significant. Combined with the analysis of RQ1, we can infer that the influence of L2 Japanese acquisition on native Chinese processing was relatively less constrained by orthographic and phonological similarities during native Chinese word-naming task. This result contrasts with previous research (e.g., Cai and Matsumi, 2009; Fei and Matsumi, 2012; Fei et al., 2022), which demonstrated a significant impact of orthographic and phonological similarities between Chinese and Japanese on L2 Japanese processing. In other words, the effect of orthographic and phonological similarities is contingent upon the processing task (i.e., whether the task involves L2 Japanese processing or native Chinese processing).

Additionally, under conditions of high L2 processing efficiency, Figure 3B reveals that the processing efficiency of J-N1 learners in their L1 vocabulary was lower than that of J-N2 learners. Conversely, under conditions of low L2 processing efficiency, the Chinese processing efficiency of J-N2 learners was lower than that of J-N1 learners. This finding highlights a complex interaction between L2 Japanese processing efficiency and L1 Chinese processing efficiency. One possible explanation for this phenomenon relates to cross-linguistic interference and L2 proficiency. For learners with higher Japanese proficiency (i.e., J-N1 learners), as their proficiency in L2 increases, their level of L2 automatization also rises (Shinozuka et al., 2021). Consequently, when the L2 processing efficiency of J-N1 learners is high, they may struggle to differentiate between the two languages during L1 processing. This difficulty may result in stronger cross-linguistic interference, ultimately reducing efficiency in L1 processing. In contrast, for learners with lower Japanese proficiency (i.e., J-N2 learners), even though their L2 processing efficiency is high, the cross-linguistic interference effect may be less pronounced, resulting in relatively faster response times during L1 processing. On the other hand, under conditions of low processing efficiency, the cross-linguistic interference effect is likely diminished for both J-N1 and J-N2 learners. At this point, for the relatively slower non-selective activation of L2, J-N1 learners exhibit a more balanced bilingual performance and demonstrate stronger inhibitory control over non-selective activation (Altamimi, 2016). In contrast, J-N2 learners, owing to their relatively lower proficiency, may exhibit weaker inhibitory control over the non-selective activation of L2, resulting in longer response times.

## 5 General discussion

This study explored the impact of L2 Japanese acquisition on native Chinese lexical processing within the context of Chinese-Japanese bilingualism. Specifically, it examined the effects of orthographic and phonological similarities between Chinese and Japanese on L1 word-naming task performance, taking into account L2 proficiency and L2 processing efficiency. This section synthesizes the results, discusses their implications, and proposes a processing model for Chinese learners of Japanese based on two-character Chinese words from the perspectives of orthography and phonology.

### 5.1 Factors influencing native lexical processing among Chinese learners of Japanese

Chinese native speakers without any prior Japanese learning experience exhibited significantly faster response times in L1 lexical naming compared to both J-N1 and J-N2 learners. This finding suggests that during the processing of native Chinese vocabulary, L2 Japanese lexical representations are automatically activated. This activation leads to competition and inhibition between the two languages, consuming more cognitive resources and resulting in longer response times. Previous studies have confirmed that bilingual activation leads to competition (Ivanova and Costa, 2008; Hsieh et al., 2017; Song et al., 2023), and this study further demonstrates that such competition not only affects L2 processing but also impacts L1 processing.

To further explore the impact of similarity between languages on bilinguals, we found that orthographic similarity between Chinese and Japanese did not significantly impact native Chinese lexical processing. Previous research has shown that orthographic similarity between Chinese and Japanese facilitates L2 Japanese lexical acquisition and processing for Chinese learners (e.g., Matsumi et al., 2012). Nonetheless, in the context of L1 processing, this similarity did not show a particularly significant effect. We believe this result may be related to the stimuli and experimental design used in this study. The stimuli used to investigate orthographic similarity were words with low phonological similarity between Chinese and Japanese. The word-naming task’s final performance was measured by phonological output response time. Regardless of the level of orthographic similarity, the phonological connection between the two languages was relatively weak, thus failing to show a significant effect. Future research could further verify this finding using different tasks.

In contrast, there was a significant interaction between phonological similarity and Japanese proficiency. It can be assumed that native Chinese speakers who have not learned Japanese are not affected by the phonological similarity between Chinese and Japanese. Using these speakers as a baseline for analysis revealed that phonological similarity was more likely to influence advanced learners (i.e., J-N1 learners). Higher phonological similarity resulted in longer response times for J-N1 learners, whereas J-N2 learners were less affected by phonological similarity in terms of L2 activation. Fei et al. (2022) confirmed that Chinese learners of Japanese experience inhibitory effects from phonological similarity during L2 Japanese processing. Our study extends this finding to L1 processing, demonstrating that phonological similarity between Chinese and Japanese impedes both L1 and L2 processing in bilinguals.

Furthermore, this study confirms that L2 proficiency and L2 processing efficiency jointly influence native Chinese lexical processing. For J-N1 learners, the correlation between L2 Japanese lexical processing efficiency and native Chinese lexical processing efficiency was lower, whereas for J-N2 learners, it was higher. These results indicate that intermediate-level Japanese learners exhibit a stronger connection between their L1 and L2, with higher L2 processing efficiency correlating with higher L1 processing efficiency. As L2 proficiency improves, the automatization level of L2 processing increases (e.g., Shinozuka et al., 2021), and bilingual processing becomes relatively independent (Jiang, 2000). However, for J-N2 learners, who experience a greater imbalance between their L1 and L2, although they rely more on their L1, the presence of non-selective activation phenomena led to bilingual competition. Especially for learners with low L2 processing efficiency, the non-selectively activated L2 cannot be processed and inhibited immediately, resulting in longer response times.

### 5.2 Native Chinese lexical processing model for Chinese learners of Japanese

**Figure 4:**
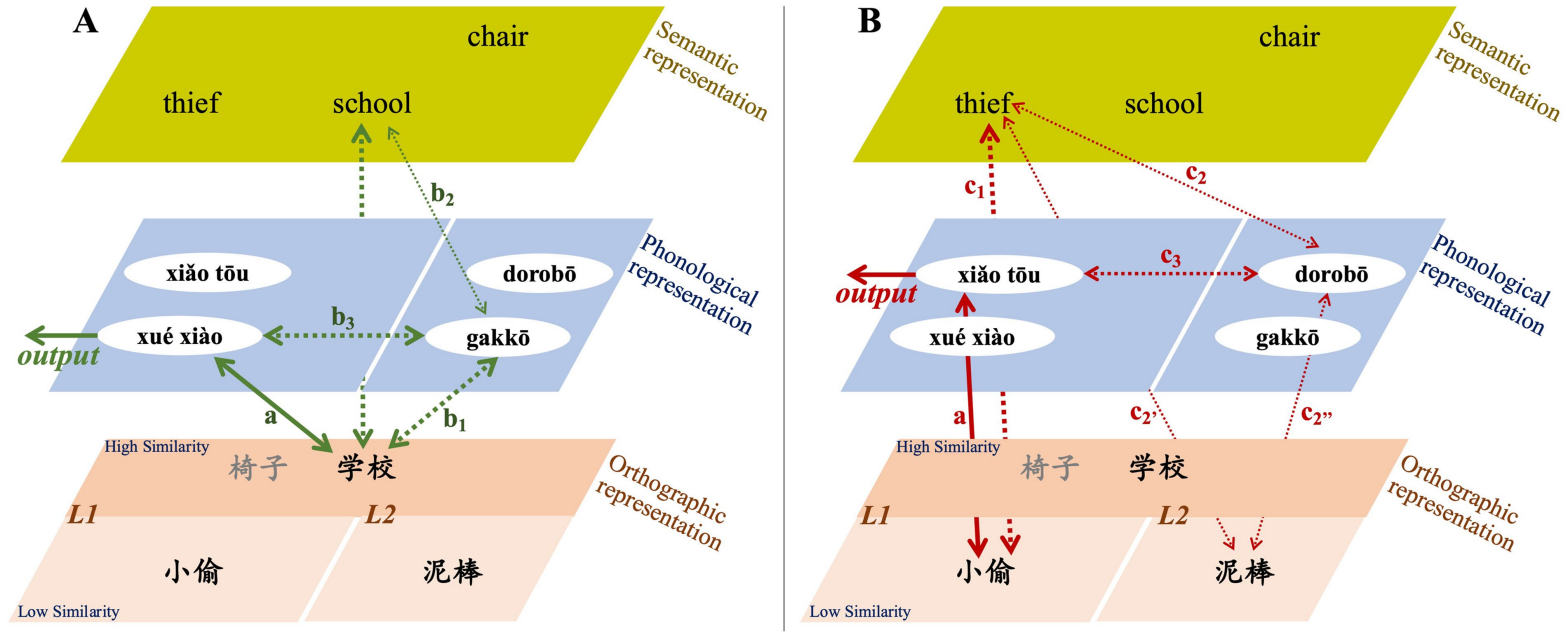
Influence pathway of orthographic similarity in L1 processing for Chinese learners of Japanese. **(A)** Illustrates the L1 processing model under the condition of high orthographic similarity, while panel **(B)** illustrates the model under the condition of low orthographic similarity. The thickness and style of the arrows represent the strength of the processing link, with thicker solid arrows indicating stronger pathways. Green arrows indicate words with high orthographic similarity and low phonological similarity, while red arrows indicate words with low orthographic similarity and low phonological similarity. The left sections represent Chinese (L1) lexical representation, while the right sections represent Japanese (L2) lexical representation. Since the experimental results did not show significant effects for orthographic similarity, we incorporated comparisons based on the magnitude of response-time differences to describe the processing model.

**Figure 5:**
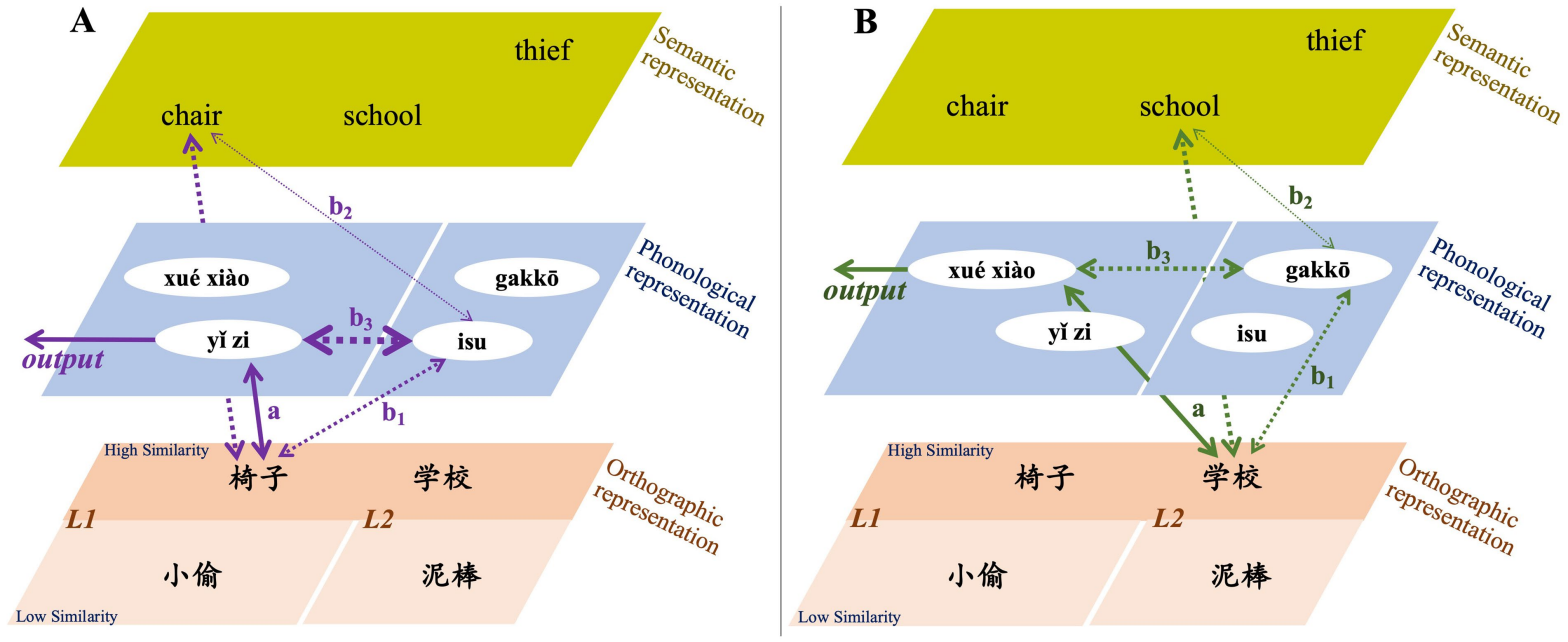
Influence pathway of phonological similarity in L1 processing for Chinese learners of Japanese. **(A)** Illustrates the L1 processing model under the condition of high phonological similarity, while panel **(B)** illustrates the model under the condition of low phonological similarity. The thickness and style of the arrows represent the strength of the processing link, with thicker solid arrows indicating stronger pathways. Purple arrows indicate words with high orthographic similarity and high phonological similarity, while green arrows indicate words with high orthographic similarity and low phonological similarity. The left sections represent Chinese (L1) lexical representation, while the right sections represent Japanese (L2) lexical representation. Since the experimental results did not show significant effects for phonological similarity, we incorporated comparisons based on the magnitude of response-time differences to describe the processing model.

Based on the experimental results, this study proposes a model for the activation of various psychological representations and the processing pathways between Chinese and Japanese when Chinese learners of Japanese, who have not studied in Japan, process their native Chinese. Following the characteristics of the word-naming task under visual input conditions, we first explore the impact of orthographic similarity (Figure 4). Subsequently, we investigate the influence of phonological similarity (Figure 5). As previously mentioned, this study used native Chinese speakers who have not learned Japanese as a reference to explore the impact of Japanese acquisition on the L1. Since the experimental results did not show significant main effects for orthographic similarity or phonological similarity, we incorporated comparisons based on the magnitude of response-time differences to describe the processing model.

Native Chinese speakers without Japanese learning experience were not affected by the interference effect of Japanese representations, allowing them to quickly complete the word-naming task (Figures 4A,B: arrow-a, i.e., *direct processing*). For Japanese learners, when they named visually presented Chinese vocabulary in their L1, the representation of L2 Japanese was activated regardless of whether the vocabulary exhibited high or low orthographic similarity between Chinese and Japanese. According to the descriptive statistics of response times, it is evident that both J-N1 and J-N2 learners had longer naming response times for Chinese words with high orthographic similarity compared to those with low orthographic similarity. This difference was particularly pronounced in J-N2 learners, especially those with low L2 Japanese processing efficiency.

Research has shown that the activation from L1 to L2 typically occurs through a conceptual association model (Kroll and Steward, 1994). Furthermore, studies have demonstrated that when using an ideographic writing system, the semantic representation is directly activated (e.g., Cai and Matsumi, 2009; Fei and Matsumi, 2012). Building on these existing findings and the results of our current study, we propose that, regardless of the level of orthographic similarity, the visual input of Chinese characters activates the L2 phonological representation. This activation leads to bilingual competition and results in prolonged response times. When words with high orthographic similarity are presented, not only is the path represented by Figure 4A: arrow-a activated, but the paths indicated by Figure 4A: arrow-b_1_ and Figure 4A: arrow-b_2_—which reflects the activation of the L2 phonological representation via semantic representation—are also activate. This activation results in competition with the L1 phonological representation (Figure 4A: arrow-b_3_). In contrast, when the visual input consists of words with low orthographic similarity, the activation of the path represented by Figure 4B: arrow-a occurs alongside the activation of the L2 phonological representation. This activation occurs solely through semantic representation as a mediator (i.e., Figure 4B: arrow-c_1_ → c_2_ or arrow-c_1_ → c_2’_ → c_2”_). As a result, competition is formed (Figure 4B: arrow-c_3_). However, the overall intensity of this semantic-mediated phonological activation is weaker than the direct phonological activation associated with words that exhibit high orthographic similarity, resulting in relatively less interference and faster response times.

Integrating the above results, it is evident that high orthographic similarity in Chinese characters leads to a relatively greater interference effect. Building on this foundation, we further explore the impact of phonological similarity. First, regardless of the level of phonological similarity, the results show that native Chinese speakers without Japanese learning experience were not affected by the interference effect of Japanese representations, allowing them to quickly complete the word-naming task (Figures 5A,B: arrow-a, i.e., *direct processing*). However, for Japanese learners, while the path represented by Figures 5A,B: arrow-a is activated, the paths indicated by Figures 5A,B: arrow-b_1_ and arrow-b_2_ are also simultaneously activated. This leads to competition with the L1 phonological representation (Figures 5A,B: arrow-b_3_). Under conditions of high phonological similarity, stronger interference effects arise due to the similarity in bilingual pronunciations (e.g., 椅子: yǐ zi vs. isu), resulting in longer response times (e.g., Fei and Matsumi, 2012; Fei et al., 2022). In contrast, for words with low phonological similarity (e.g., 学校: xué xiào vs. gakko), even though both bilingual phonological representations are activated, the interference effects are smaller, resulting in overall faster response times. Moreover, the analysis of interaction effects reveals that J-N1 learners were more significantly affected by phonological similarity, whereas J-N2 learners experienced relatively less impact. This suggests that phonological similarity has a more pronounced interference effect on learners with higher proficiency in Japanese, compared to those with lower proficiency.

## 6 Conclusions and limitations

### 6.1 Conclusion

Building on existing research that examines the influence of L1 on L2 acquisition and processing, this study investigated the reverse perspective: the impact of L2 acquisition on L1 processing. By manipulating orthographic and phonological similarities between Chinese and Japanese, and considering learners’ stages of learning and L2 processing efficiency, a series of experiments were conducted. The main conclusions of this study are as follows:

First, the acquisition of L2 Japanese significantly influences native Chinese lexical processing for Chinese learners of Japanese. Second, although there are indications of orthographic and phonological similarities between Chinese and Japanese affecting native Chinese processing, the overall strength of these effects appears to be relatively weak. Third, the impact of L2 proficiency on L1 lexical processing is also influenced by L2 lexical processing efficiency, and both factors are closely related, jointly affecting the lexical processing pathway.

### 6.2 Limitations and future research

First, this study only utilized oral response times to investigate the research results. Although we are aware of the potential limitations associated with using response time as a measure in psychological experiments (see Crocetta and Andrade, 2015), and we controlled experimental conditions as much as possible, more metrics are needed for a multidimensional discussion of the research conclusions. In the future, eye-tracking studies and EEG measures could be employed to further explore the impact of L2 acquisition on L1 processing from a comparative analysis perspective.

Second, the participants in this study were Chinese university students learning Japanese in classrooms, with no long-term residency experience in Japan. Future research could extend the subject pool to include Chinese students studying in Japan and Japanese learners of Chinese. This would provide a more comprehensive examination of the impact of L2 acquisition on L1 processing.

Lastly, this study focused only on orthographic and phonological similarities. Future research could further explore semantic processing, combining experimental paradigms such as semantic processing tasks and auditory priming experiments to comprehensively investigate the interactions between L2 and L1.

## Data Availability

The raw data supporting the conclusions of this article will be made available by the authors, without undue reservation.

## Footnotes

^1^N2 learners typically have approximately 600 to 800 h of study time, enabling them to understand coherent conversations or news spoken at near-natural speed in everyday situations. They are also able to comprehend logically complex texts, such as newspapers or straightforward critiques, and express opinions on general topics (Kouno and Hashimoto, 2016). The J-N2 learners in this study followed a course load that included grammar and vocabulary instruction (6 h per week over 32 weeks for two academic years), Japanese listening (3 h per week over 32 weeks for two academic years), reading (1.5 h per week over 32 weeks for one academic year), and writing (1.5 h per week over 32 weeks for one academic year), totaling approximately 700 h of study. To further examine differences in Japanese processing abilities between J-N1 and J-N2 learners, we calculated the average response time for Japanese lexical processing for each participant and conducted a group comparison. The results showed a significant difference between the two groups, with J-N1 learners exhibiting significantly shorter response times compared to J-N2 learners [*t* (36) = 3.32, *p* = 0.002]. This finding suggests that N1 learners have higher processing efficiency, which, to a certain extent, supports the validity of the group classifications in this study.
