# Supplementary material for: Japanese-as-a-foreign-language acquisition affects native Chinese lexical processing among Chinese learners
Source: Front Psychol. 2025 Jan 7;15:1457155. doi: 10.3389/fpsyg.2024.1457155 (PMC11756525; doi:10.3389/fpsyg.2024.1457155)
Supplement: Supplementary file 1 [file Data_Sheet_1.pdf]

Supplementary Material

1 Experimental Materials

	Stimuli			*Log-transformed frequency*		*Familiarity*		*Phonological similarity*
	Chinese	*Japanese*	*English translation*	*Chinese*	*Japanese*	*N1 learners*	*N2 learners*	
Condition 1: high orthographic similarity; high phonological similarity								
1	椅子	椅子	chair	4.47	3.68	6.76	6.73	5.62
2	电话	電話	telephone	5.41	4.47	6.90	6.86	5.38
3	空气	空気	air	4.89	3.79	6.86	6.55	4.42
4	住所	住所	residence	3.83	3.72	6.52	6.55	6.69
5	失败	失敗	failure	4.81	3.79	6.90	6.55	6.27
6	安心	安心	relief	4.34	3.84	6.71	6.59	6.96
7	理由	理由	reason	4.93	4.29	6.90	6.77	6.31
8	出发	出発	departure	4.93	3.69	6.86	6.86	4.00
9	散步	散歩	walk	4.16	3.51	6.86	6.82	4.65
10	教室	教室	classroom	4.51	3.82	6.86	6.82	4.85
11	热心	熱心	enthusiasm	4.18	3.32	6.76	6.68	3.85
12	时代	時代	era	5.22	4.55	6.48	5.82	5.15
13	日记	日記	diary	4.34	3.55	6.43	6.23	4.69
14	练习	練習	practice	4.64	3.83	6.90	6.82	6.42
15	漫画	漫画	comic	4.27	3.30	6.86	6.77	4.73
Condition 2: high orthographic similarity; low phonological similarity								
1	学校	学校	school	5.63	4.58	6.90	6.86	1.54
2	交通	交通	transportation	5.25	4.14	6.67	6.82	2.58
3	预约	予約	reservation	3.87	3.75	6.76	6.73	1.69
4	音乐	音楽	music	5.14	4.05	6.71	6.77	1.69
5	旅行	旅行	travel	4.74	3.97	6.81	6.82	3.04
6	生活	生活	life	5.83	4.62	6.81	6.77	2.00
7	说明	説明	explanation	5.23	4.37	6.24	5.68	1.23
8	入学	入学	enrollment	4.26	3.46	6.90	6.77	1.50
9	作文	作文	writing	4.27	2.78	6.67	6.82	1.92
10	医学	医学	medicine	4.98	3.64	6.24	6.50	2.88
11	地图	地図	map	4.28	3.60	6.71	6.59	2.35

12	数学	数学	mathematics	4.76	3.41	6.38	6.59	1.73
13	牛肉	牛肉	beef	4.37	3.16	6.52	6.64	2.69
14	铅笔	鉛筆	pencil	3.89	2.94	6.71	6.50	2.00
15	特别	特別	special	5.58	4.19	6.76	6.73	1.46
Condition 3: low orthographic similarity; low phonological similarity								
1	小偷	泥棒	thief	4.11	2.95	6.33	5.95	1.65
2	小孩	子供	child	4.90	4.48	6.05	6.32	1.85
3	物品	品物	item	4.43	3.10	6.52	6.45	2.15
4	建筑	建物	building	5.32	3.92	6.57	6.55	1.00
5	情形	具合	situation	4.65	3.53	6.33	6.27	2.15
6	比赛	試合	competition	5.26	3.86	6.76	6.77	2.65
7	水果	果物	fruit	4.61	3.06	6.86	6.82	1.35
8	钟表	時計	watch	3.39	3.67	6.90	6.73	1.69
9	介绍	紹介	introduction	5.30	4.16	6.90	6.82	2.27
10	吃饭	食事	meal	5.07	4.02	6.76	6.82	1.46
11	手套	手袋	glove	4.10	2.92	6.00	6.09	1.15
12	名字	名前	name	5.09	4.20	6.86	6.82	1.12
13	节目	番組	show	4.88	3.82	6.57	6.68	2.15
14	答复	返事	reply	4.10	3.66	6.90	6.55	2.58
15	房间	部屋	room	4.97	4.30	6.86	6.36	1.15
